# *In vivo* Conditions Induce Faithful Encoding of Stimuli by Reducing Nonlinear Synchronization in Vestibular Sensory Neurons

**DOI:** 10.1371/journal.pcbi.1002120

**Published:** 2011-07-21

**Authors:** Adam D. Schneider, Kathleen E. Cullen, Maurice J. Chacron

**Affiliations:** 1 Department of Physics, McGill University, Montreal, Quebec, Canada; 2 Department of Physiology, McGill University, Montreal, Quebec, Canada; École Normale Supérieure, College de France, CNRS, France

## Abstract

Previous studies have shown that neurons within the vestibular nuclei (VN) can faithfully encode the time course of sensory input through changes in firing rate *in vivo*. However, studies performed *in vitro* have shown that these same VN neurons often display nonlinear synchronization (i.e. phase locking) in their spiking activity to the local maxima of sensory input, thereby severely limiting their capacity for faithful encoding of said input through changes in firing rate. We investigated this apparent discrepancy by studying the effects of *in vivo* conditions on VN neuron activity *in vitro* using a simple, physiologically based, model of cellular dynamics. We found that membrane potential oscillations were evoked both in response to step and zap current injection for a wide range of channel conductance values. These oscillations gave rise to a resonance in the spiking activity that causes synchronization to sinusoidal current injection at frequencies below 25 Hz. We hypothesized that the apparent discrepancy between VN response dynamics measured in *in vitro* conditions (i.e., consistent with our modeling results) and the dynamics measured *in vivo* conditions could be explained by an increase in trial-to-trial variability under *in vivo* vs. *in vitro* conditions. Accordingly, we mimicked more physiologically realistic conditions in our model by introducing a noise current to match the levels of resting discharge variability seen *in vivo* as quantified by the coefficient of variation (CV). While low noise intensities corresponding to CV values in the range 0.04–0.24 only eliminated synchronization for low (<8 Hz) frequency stimulation but not high (>12 Hz) frequency stimulation, higher noise intensities corresponding to CV values in the range 0.5–0.7 almost completely eliminated synchronization for all frequencies. Our results thus predict that, under natural (i.e. *in vivo*) conditions, the vestibular system uses increased variability to promote fidelity of encoding by single neurons. This prediction can be tested experimentally *in vitro*.

## Author Summary

The vestibular system senses the motion of the head in space and is vital for gaze stability, posture control, and the computation of spatial orientation during everyday life. The activities of single vestibular neurons recorded in the brains of awake behaving animals show that they can accurately transmit information about the time course of head motion, which is necessary for several behaviors such as the vestibulo-ocular reflex required for gaze stabilization. In contrast, this is not the case when the same neurons are recorded in isolation and sensory stimulation is mimicked experimentally. We investigated the cause for this discrepancy by studying how a mathematical model of vestibular neuron activity responds to mimics of sensory stimulation under different conditions. We found that the differences in the activities of vestibular neurons recorded in awake behaving animals and in isolation can be explained by the addition of synaptic noise, which in turn, increases the variability of action potential firing that is seen in more natural conditions. Our modeling results make a clear prediction that can be tested experimentally.

## Introduction

The vestibular system provides information about head motion relative to space that is necessary for maintaining posture, computing spatial orientation, and perceiving self-motion. Peripheral vestibular afferents encode the detailed time course of either horizontal rotations, vertical rotations, or linear acceleration through changes in their firing rates and spike timing [1]–[4]. These afferents project unto neurons within the vestibular nuclei (VN) [5]–[7]. *In vitro* studies have established that VN neurons in mammals are classified into two main subpopulations (type A and type B) that differ in their responses to current input as well as action potential shape [8]–[11]. In response to depolarizing current steps, type A neurons show a sustained tonic response while the type B neurons display spike frequency adaptation. Type B neurons moreover display a resonance at frequencies within the behaviorally relevant range that increases the tendency of small amplitude, high-frequency synaptic inputs to trigger non-linear firing behavior in the form of synchronization to the peaks of the input [12], [13]. This synchronization severely limits the range of input frequencies and amplitudes for which the activity of type B neurons accurately follows the input [13]–[15]. In contrast, type A neurons, despite also displaying a resonance, tend to follow the time course of current injection accurately for a much wider range of stimulus amplitudes [12], [13].

In contrast, the results of *in vivo* experiments have shown that the firing of many VN neurons accurately follows the time course of sensory stimulation over the behaviorally relevant frequency range (0–20 Hz) [16], [17]. While this result is at odds with those of *in vitro* studies, it is consistent with the fact that eye movement produced by the vestibuloocular reflex (VOR), which is largely driven by the activities of VN neurons, has a very short latency and is accurate over this same frequency range [18], [19]. How can the same neurons display nonlinear responses such as synchronization *in vitro* and yet accurately follow the time course of sensory input *in vivo*? The discrepancy can be dramatic. For example, Floccular target neurons (FTNs) have been shown to correspond to a subpopulation of type B VN neurons [20], [21] that display the strongest tendency for nonlinear synchronization *in vitro*, yet do not display such synchronization in response to sensory input *in vivo* [16].

Here we test the hypothesis that the apparent discrepancy between VN response dynamics in the *in vitro* and *in vivo* conditions can be explained by an increase in trial-to-trial variability under *in vivo* vs. *in vitro* conditions. To do so, we used a simplified biophysical model that has been previously used to describe VN neuron activity *in vitro* [14]. We show that this model displays membrane potential oscillations that give rise to a resonance in the membrane potential response. This resonance is transferred to the spiking response and causes nonlinear synchronization to sinusoidal current injections over a wide range of frequencies (0–20 Hz). We then mimicked the high-conductance state that is typical of *in vivo* conditions in our model by increasing the membrane conductance. Moreover, we mimicked their large resting discharge rates by increasing the bias current. Interestingly, both of these changes in parameter values were not sufficient to remove this synchronization that thus severely limits the range of inputs for which our model's response follows the input accurately. However, we show that adding noise to our model in order to mimic the resting discharge variability displayed by VN neurons *in vivo* can be sufficient to eliminate synchronization over the full range of behaviorally relevant frequencies.

## Results

**Figure 1:**
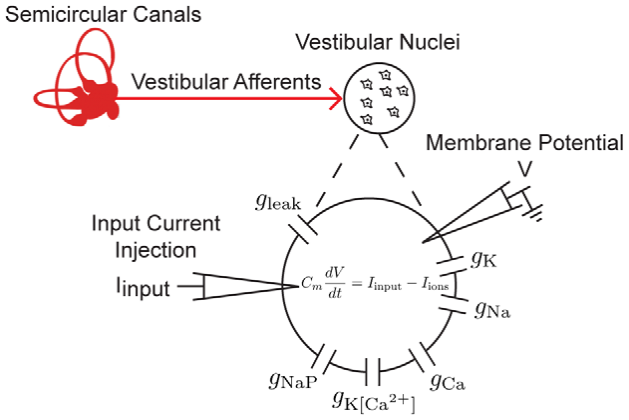
Vestibular anatomy and model description. Schematic of peripheral vestibular system, indicating projections from semi-circular canals to the vestibular nuclei (VN). VN neurons were modeled using the Hodgkin-Huxley formalism with several membrane conductances as shown. Sensory input was mimicked by somatic current injection.

Our biophysical model is based on the Hodgkin-Huxley formalism and consists of a single compartment endowed with several membrane conductances (see Methods and Figure 1). Note that a full biophysical justification of the model can be found elsewhere [12], [14]. Although previous studies have shown that this model could display a resonance in its spiking response to sinusoidal current injections [14], they have not systematically explored its dependence on different parameters as well as the interactions between different membrane conductances that underlies its generation.

As it has been previously shown that resonances in the spiking response could be caused by resonances in the membrane potential [22], we first investigated the models capacity to display membrane potential oscillations in response to current input. To do so, we first turned off the spiking sodium and rectifier potassium conductances by setting their maximum conductances values to zero (i.e. 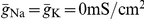). We note that this approach is valid for the parameter values used here (see Methods).

### Intrinsic membrane conductances give rise to damped membrane potential oscillations in the presence of perturbations

It is well known that damped or sustained membrane potential oscillations can arise from the interplay between several membrane conductances including voltage gated calcium channels [23]. The magnitude of these oscillations is furthermore strongly dependent on the amount of depolarizing current bias [22]. As such, we varied both the maximum calcium conductance 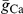 and the bias current 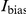 in our model. We first studied the membrane potential response to step current injections as these have been previously used to demonstrate the presence of membrane potential oscillations [23].

**Figure 2:**
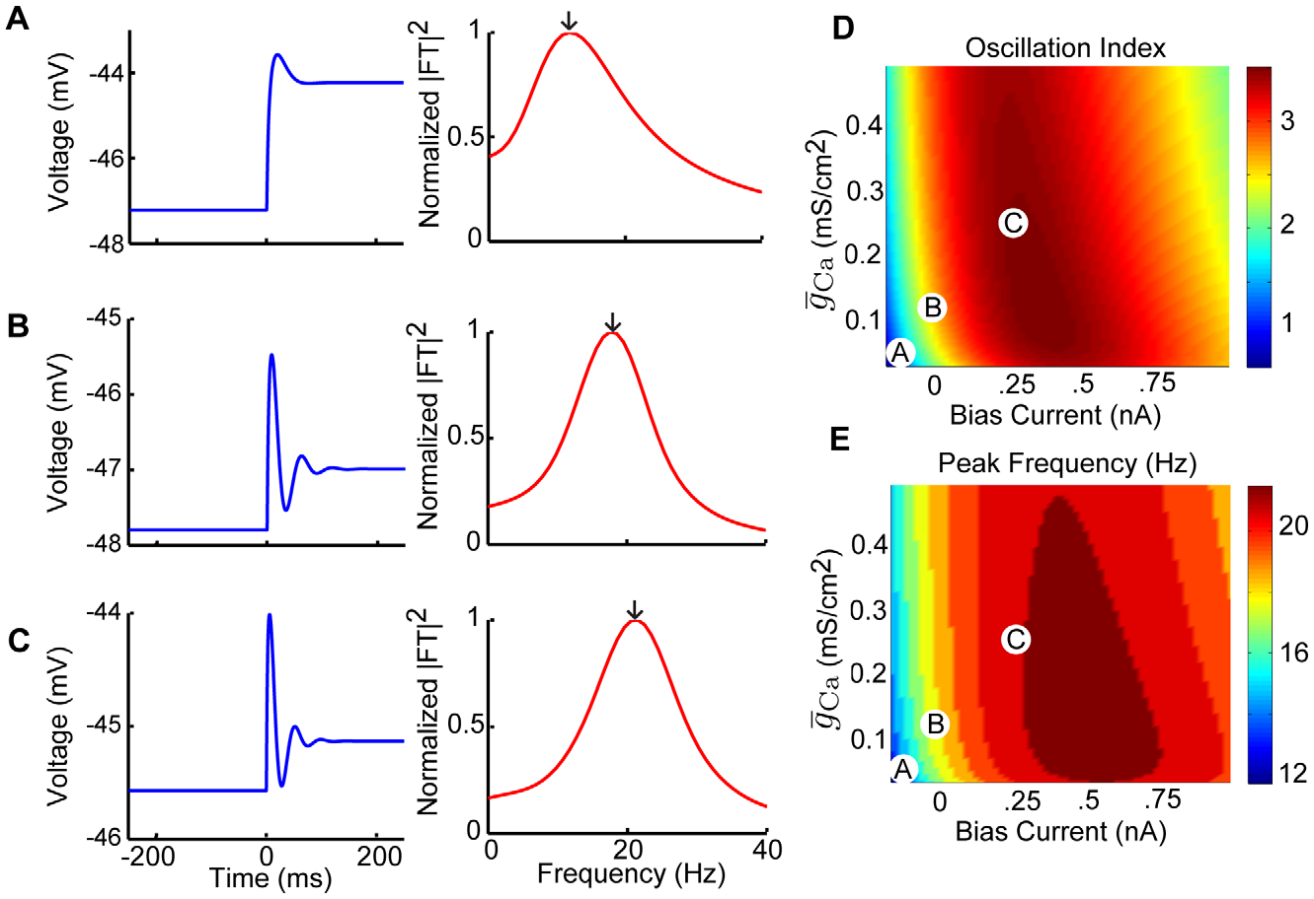
The model displays damped membrane potential oscillations in response to step current input. The model's membrane potential response to step current input was characterized for a physiologically plausible range of bias current and calcium conductance values. A–C) Example membrane voltage responses and the normalized squared magnitude of their Fourier transforms. These correspond to parameter values as follows: A) 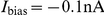, 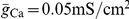, B) 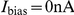, 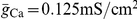, and C) 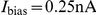, 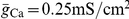. D) Oscillation index (see Methods) measuring the strength of the oscillation in the subthreshold response as a function of 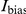 and 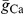. E) The peak frequency component of the squared magnitude of the responsesÕ Fourier transforms as a function of 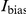 and 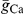. The parameter values corresponding to panels A,B,C are also shown. Other parameter values were: 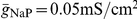, 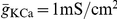, and 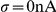.

Our results show that the model can display damped membrane potential oscillations with different magnitudes and frequencies for a wide range of 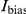 and 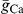 values (Figures 2A,B,C). We characterized this dependency by systematically varying both 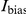 and 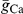 over a wide range of values and quantified the amplitude of these damped oscillations by computing an oscillation index (see Methods). Further, we computed the oscillation frequency from the squared magnitude of the Fourier transform of the response (see Methods). Our results show that, for a given value of the maximum calcium conductance 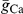, the oscillation index displays a maximum as a function of the bias current 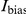 (Figure 2D). The oscillation frequency displayed qualitatively similar behavior to that of the oscillation index (Figure 2E). We note that the oscillation frequency was mostly within the behaviorally relevant range found in natural vestibular stimuli (0–20 Hz) [24]. This indicates that the model can display calcium induced damped membrane potential oscillations, the magnitude and frequency of which are highly dependent on the level of depolarizing bias current 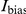. We note that qualitatively similar results were obtained when varying the persistent sodium conductance 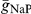 (Figure S1). The results agree with the known effects of persistent sodium, namely to depolarize the membrane and amplify the resonant behavior [23].

**Figure 3:**
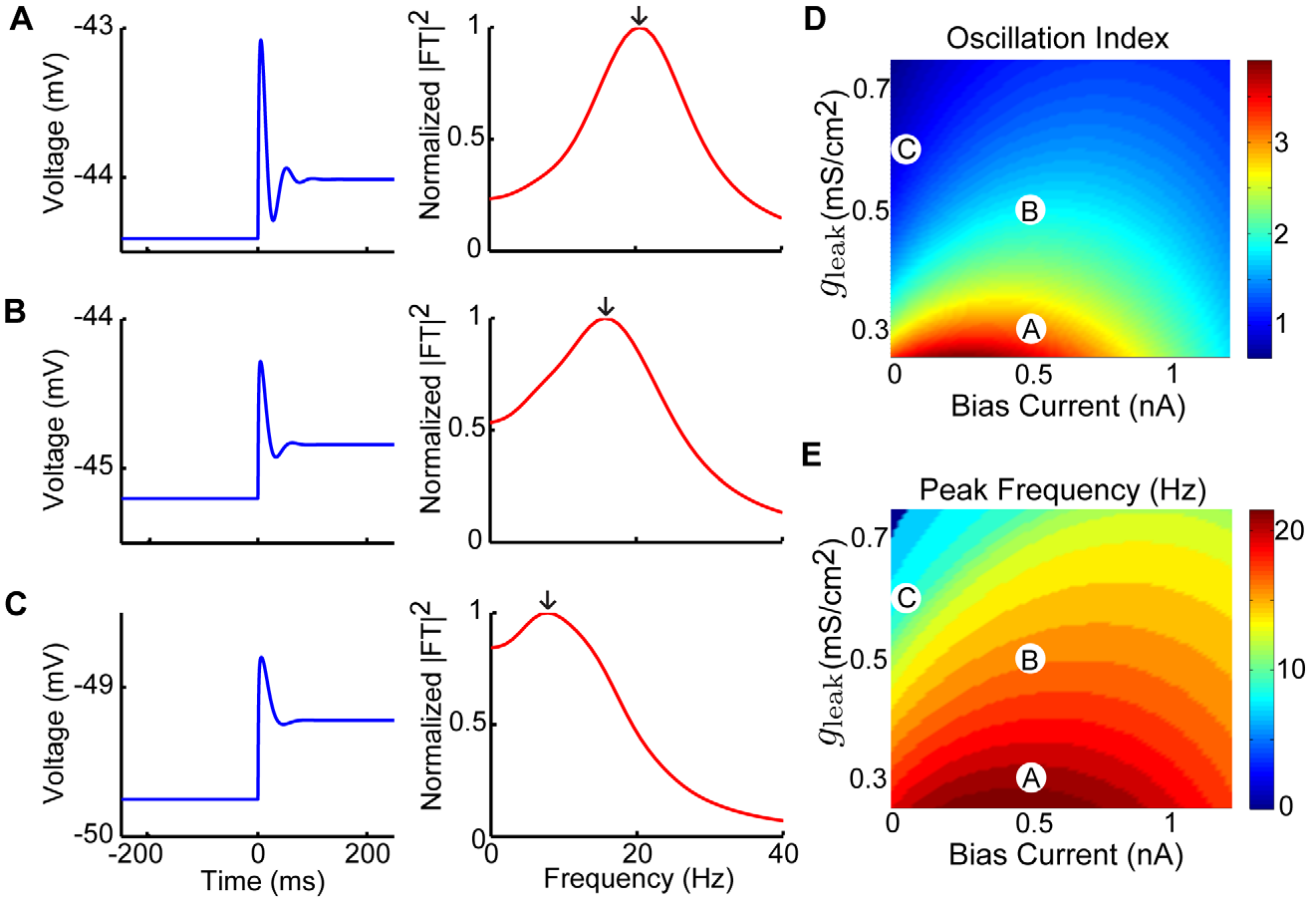
Effects of increased leak conductance on membrane potential oscillations. The model's membrane potential response to step current input was characterized for physiologically plausible ranges of bias current and leak conductance values. A–C) Example responses and the squared magnitude of their Fourier transforms. These correspond to parameter values as follows: A) 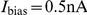, 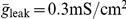, B) 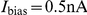, 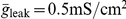, and C) 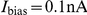, 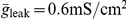. D) Oscillation index as a function of 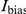 and 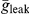. E) The peak frequency component of squared magnitude of the responses Fourier transform as a function of 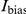 and 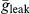. The parameter values corresponding to panels A,B,C are also shown. Other parameter values were 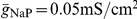, 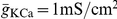, 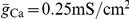, and 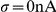.

It is well known that neurons receive massive synaptic bombardment under *in vivo* conditions, which gives rise to a high-conductance state [25], [26]. Mathematically, the increased membrane conductance under such synaptic bombardment can be mimicked by increasing the leak conductance 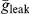 and by adding an appropriate amount of bias current [22], [27]. As such, we characterized the oscillation index and frequency as a function of both the leak conductance 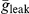 and the bias current 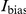. Although increasing the leak conductance 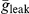 decreased the oscillation amplitude, it also decreased the oscillation frequency to values that were contained within the behaviorally relevant frequency range (Figures 3A,B,C). These changes were furthermore seen for a wide range of bias current 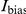 values. We observed that the oscillation index decreased as a function of the leak conductance 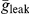 for a given value of 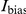 (Figure 3D). In contrast, the oscillation index displayed a maximum as a function of 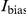 for a given value of 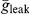 (Figure 3D). The oscillation frequency again displayed qualitatively similar behavior to that of the oscillation index as a function of both 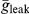 and 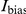 and remained within the behaviorally relevant range (Figure 3E). As such, we conclude that an increased leak conductance is not sufficient to eliminate our models tendency to display membrane potential oscillations. These oscillations could potentially be detrimental to the models ability to accurately encode the timecourse of current injections as their frequency is within the behaviorally relevant range. In order to better understand the source of these oscillations, we performed a standard perturbation analysis in our model around the resting membrane potential (see Methods). Our results show that the linearized model gave rise to oscillation indices and frequencies that were quantitatively similar to those obtained with the full model (compare Figures 2,3 with Figure S2). Moreover, computing the eigenvalues of the Jacobian matrix of the linearized system revealed that they all had a negative real part. As such, the membrane potential oscillations are unstable as our model has a stable fixed point. This is consistent with the damped oscillations that we observed in response to steps (Figure 2).

### Membrane potential oscillations induce a resonance in the spiking activity

**Figure 4:**
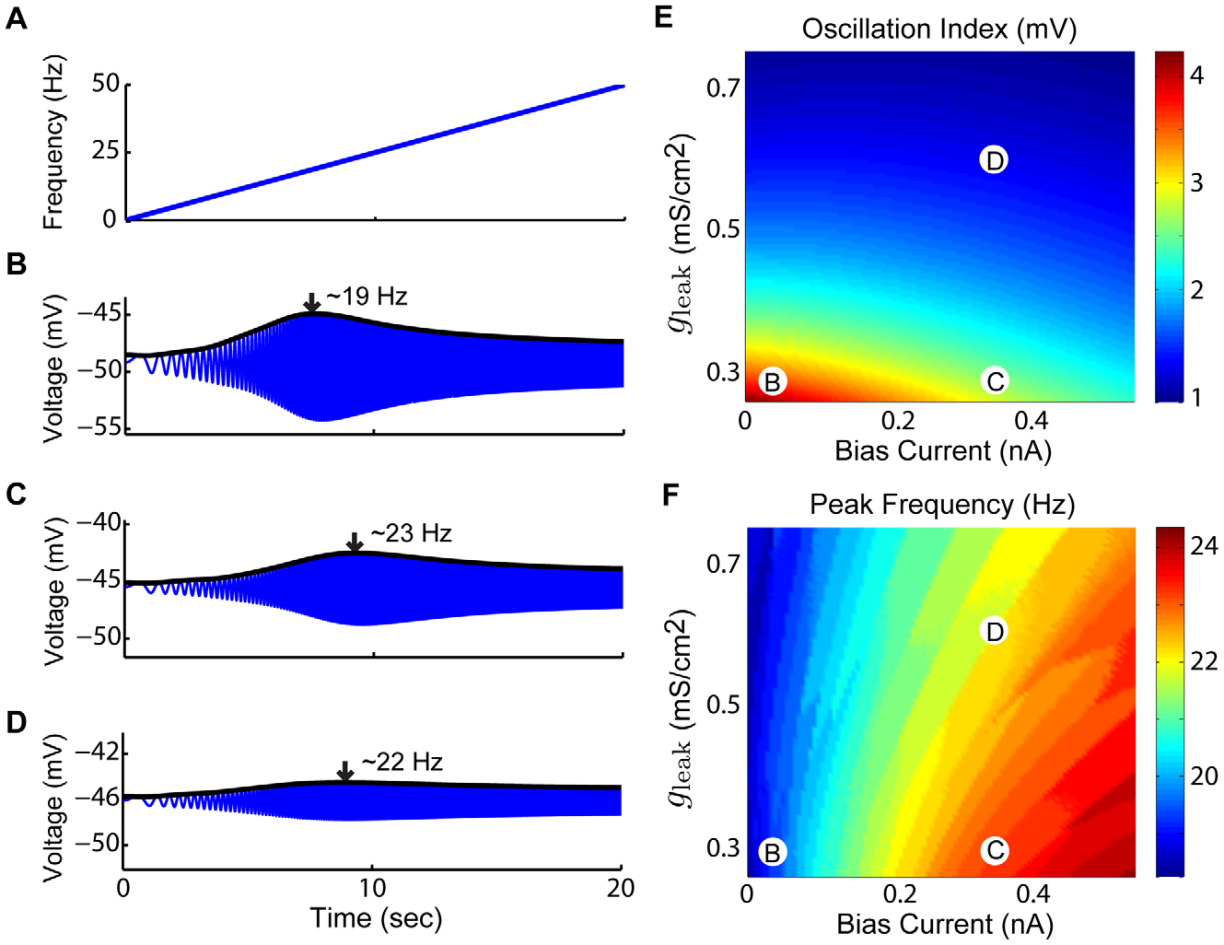
Membrane potential responses to zap current input are greatest for a given frequency. The model's membrane potential response to zap currents is greatest for a given input frequency. The magnitude of the response and the input frequency for which it occurs vary with both 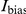 and 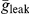. A) Instantaneous frequency of the zap stimulus frequency as a function of time. B–D) Example membrane voltage responses as a function of time, corresponding to parameter values as follows: B) 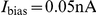, 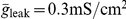, C) 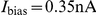, 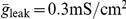, and D) 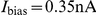, 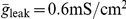. The envelope of each response is fit with a black curve with an arrow marking the peak in the response and the associated instantaneous frequency. E) Oscillation index (see Methods) as a function of 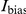 and 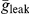. F) Oscillation frequency as a function of 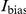 and 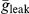. The parameter values corresponding to panels B,C,D are also shown. Other parameters values were 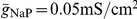 and 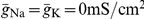.

We next investigated whether the membrane potential oscillations induced a resonance in the membrane potential response and whether this resonance causes a resonance in the spiking activity. As such, we used a zap stimulus (i.e. a sinusoidal waveform with a constant amplitude and a frequency that increases linearly as a function of time; Figure 4A) as an input to our model. Such inputs are frequently used to characterize resonant behavior [28], [29]. Our results show that the model does display a resonance in the membrane potential in response to zap current injection for different values of 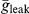 and 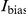 (Figures 4B,C,D). We note that these responses show asymmetries, which is to be expected since we are using a nonlinear model. We characterized this resonance by an oscillation index that quantifies its magnitude (see Methods) as well as its frequency (i.e. the zap frequency for which the membrane potential oscillation is maximal). Our results show that both the oscillation index and frequency computed from the models response to zap currents had qualitatively similar dependencies on 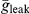 and 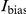 to those of the oscillation index and frequency computed from the models response to step currents (compare Figures 4E,F to Figures 3D,E, respectively).

**Figure 5:**
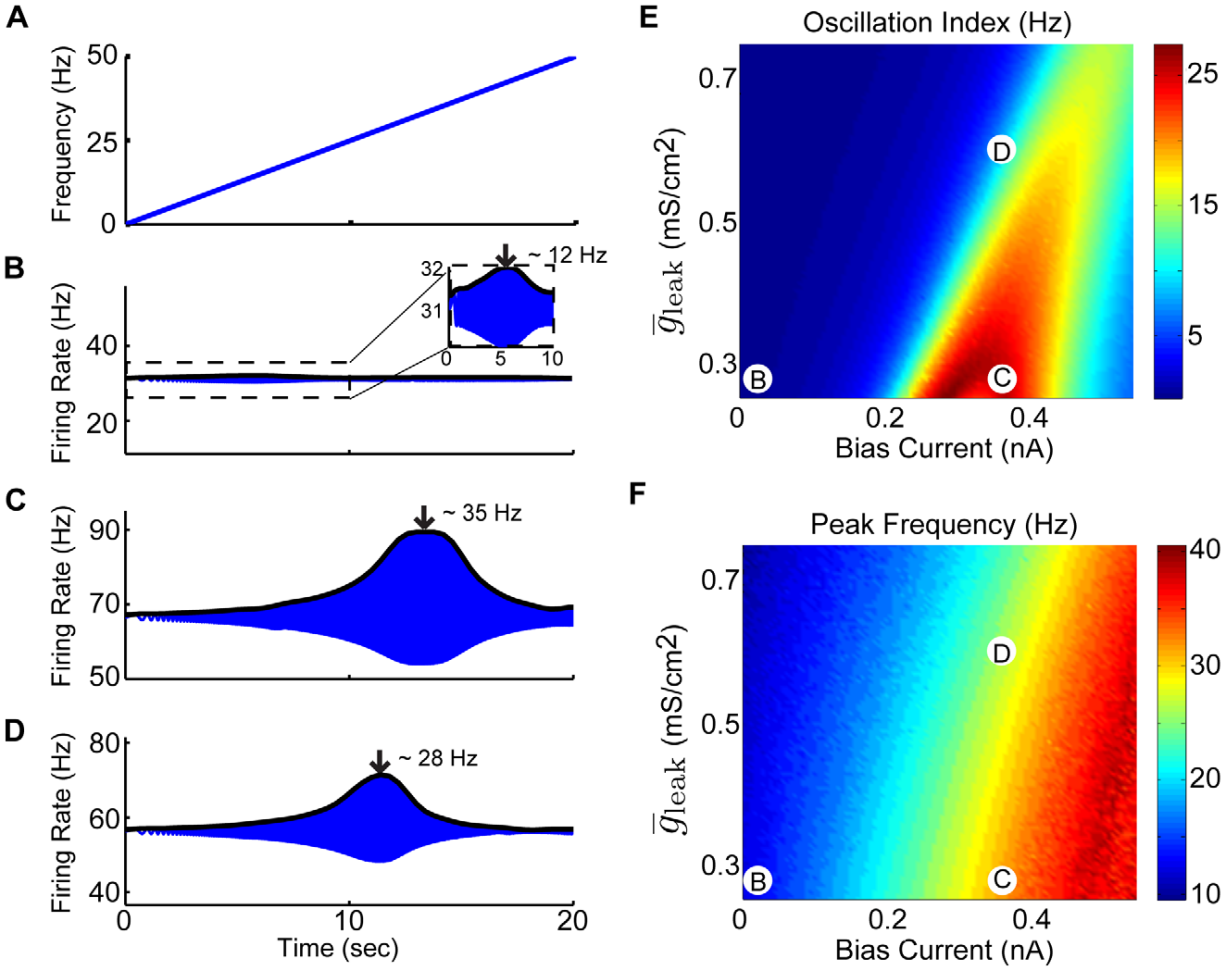
Spiking responses to zap current input display a resonance. The model's spiking response to zap current input also displays a resonance whose intensity and frequency vary with both 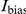 and 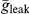. A) Instantaneous stimulus frequency as a function of time. B–D) Example instantaneous firing rates as a function of time. These correspond to parameter values as follows: B) 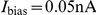, 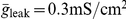, C) 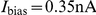, 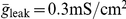, and D) 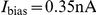, 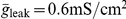. The envelope of the response is fit with a black curve with an arrow marking the location of the maximum response amplitude. E) Oscillation index as a function of 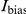 and 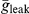. F) Oscillation frequency as a function of 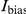 and 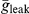. The parameter values corresponding to panels B,C,D are also shown. All other parameters had the same values as previously described except 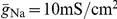 and 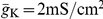.

How does resonant behavior in the membrane potential relate to resonant behavior in the spiking activity? We investigated this by turning on the spiking conductances (i.e. 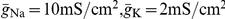) and by studying the variations in the instantaneous firing rate in response to zap current injection. Our model displayed differential resonant behavior in its spiking activity in its response to zap current injection as a function of the leak conductance 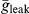 and the bias current 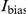 (Figures 5A, B,C,D). We note that these responses also show asymmetries, which is to be expected since we are using a nonlinear model. In general, parameter values that gave rise to resonance in the membrane potential also gave rise to resonance in the spiking activity (compare Figures 4B,C,D with Figures 5B,C,D, respectively). We further characterized the resonance in the spiking activity by an oscillation index that quantifies its magnitude (see Methods) as well as its frequency (i.e. the zap frequency for which the ensuing variation in the instantaneous firing rate is maximal). Our results show that the oscillation index and frequency computed from the spiking activity had dependencies on 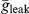 and 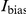 that followed qualitatively similar trends to those of the oscillation index and frequency computed from the membrane potential (compare Figures 5E,F to Figures 4E,F, respectively). Note, however, that the spiking resonance frequency varied over a wider range than the membrane potential resonance. Importantly, the resonance in the spiking regime persisted over a wide range of parameter values and its frequency overlapped with the behaviorally relevant range.

### Increasing variability promotes faithful encoding of the stimulus time course through changes in firing rate

**Figure 6:**
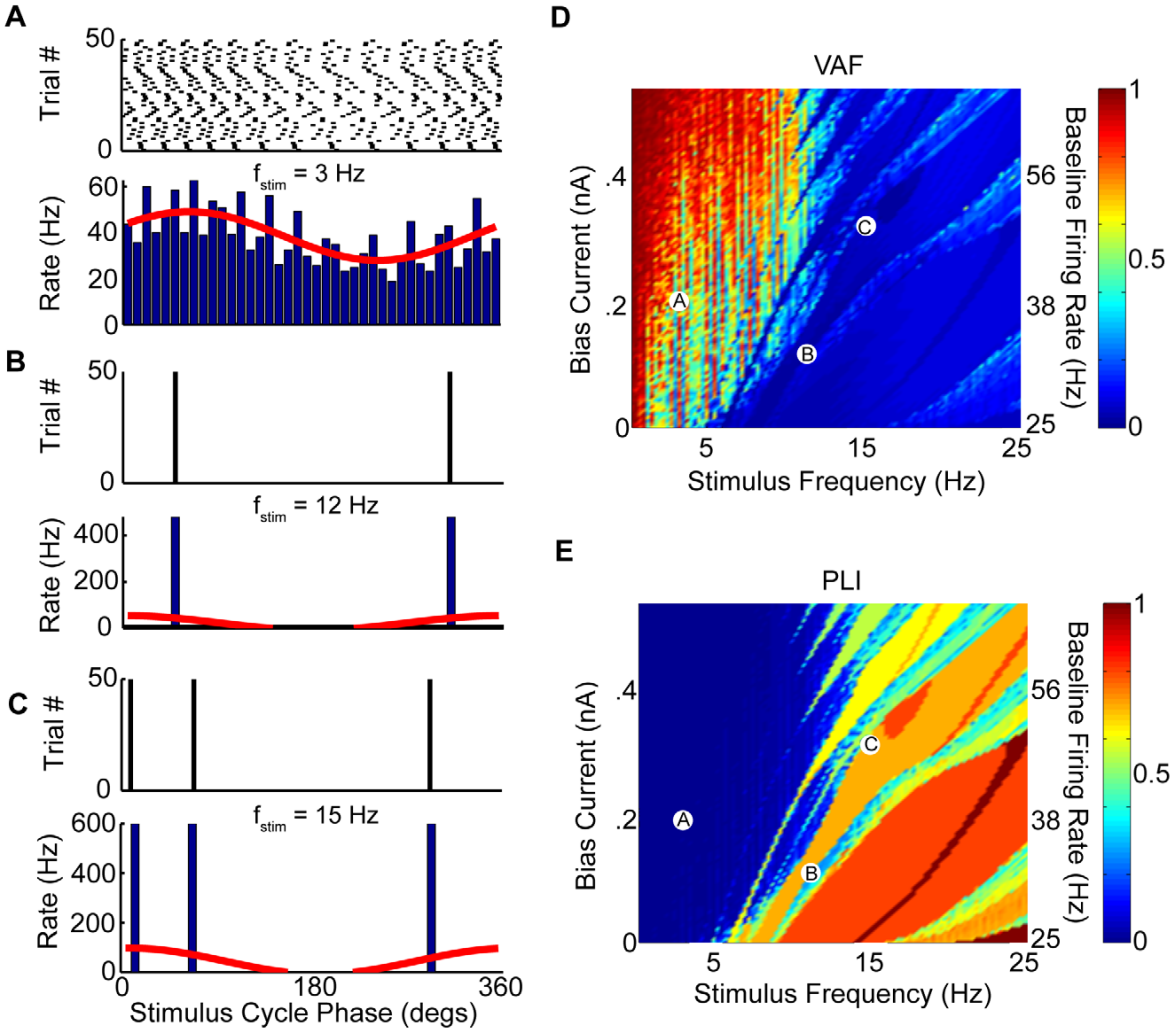
Synchronization to sinusoidal input and its consequences on faithful encoding of this input through changes in firing rate. We characterized the model's response to sinusoidal current injections with different frequencies using the phase histogram. A–C) Three example raster plots (top) and phase histograms (bottom) for different values of 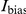 and 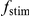. These correspond to parameter values as follows: A) 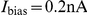, 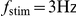, B) 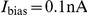, 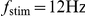, and C) 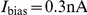, 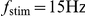. Also shown are the best fit sinusoidal curve to each phase histogram (red). D) Variance accounted for (VAF) as a function of 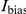 and 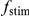. E) Phase locking index (PLI) characterizing the model's tendency to synchronize to the sinusoidal current as a function of 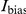 and 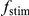. It is seen that the VAF is low for parameters for which the PLI is high and vice-versa. The parameter values corresponding to panels A,B,C are also shown. Additional parameters were the same as described previously except 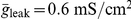.

It is expected that the resonance in the spiking activity will lead to nonlinear synchronization of the response with the peaks of the input current that is expected to be detrimental to the faithful encoding of the stimulus time course through changes in firing rate. This synchronization occurs because of the tendency of excitable systems to display n:m phase locking (i.e. fire n spikes per m cycles of forcing) in response to sinusoidal stimuli [30]–[32]. We thus characterized the models response to sinusoidal current injections that mimicked the waveforms of sinusoidal sensory stimuli used experimentally *in vivo* [16], [17], [19], [24], [33]–[36] and systematically varied the frequency of stimulation between 0 and 25 Hz. Our results show that the model tends to display phase locking for high (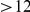 Hz) frequencies (Figures 6A,B,C). We therefore quantified the models accuracy at encoding the detailed time course of sinusoidal current injections through changes in firing rate by computing the variance accounted for (VAF, see Methods). Our results show that the VAF was high (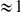) for a wide range of 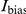 values and stimulus frequencies below 5 Hz indicating a strong tendency for faithful encoding of the current stimulus time course (Figure 6D). Increasing the baseline firing rate by increasing the bias current widened the range of stimulus frequencies for which our model displayed negligible phase locking and could faithfully encode the detailed time course of sinusoidal input from 0–5 Hz to 0–10 Hz (Figure 6D). However, we observed low VAF values (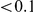) for stimulus frequencies above 10 Hz for a wide range of 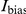 values. In order to test whether these low VAF values corresponded to parameter regimes for which our model displays phase locking, we computed a phase locking index (PLI) (see Methods). As expected, we observed that parameter regimes that gave rise to high VAF also gave rise to low PLI values and vice-versa (compare Figures 6D and 6E). This strong negative correlation between PLI and VAF for a wide range of 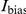 and stimulus frequencies within the natural frequency range (0–20 Hz) shows that the low VAF values correspond to a strong tendency for phase locking.

**Figure 7:**
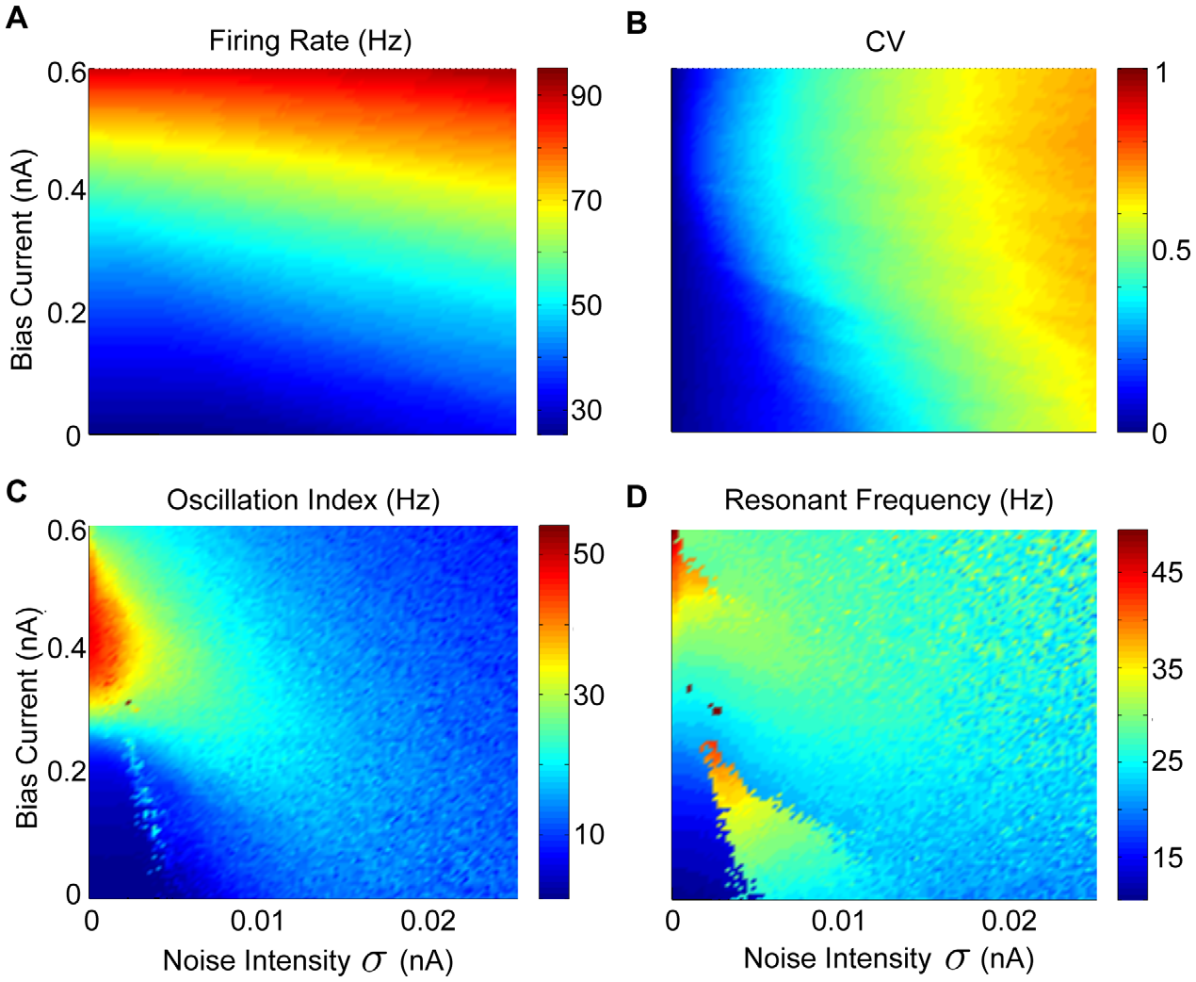
Effects of the bias current and noise intensity on resting discharge rate and variability, and resonance strength and frequency. The effects of the bias current 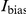 and noise intensity 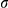 on the resting discharge rate and variability as quantified by the coefficient of variation (CV) were explored. A) Resting discharge rate as a function of 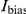 and 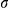. B) CV as a function of 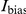 and 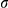. Parameter values were the same as those previously described. C) Oscillation index from zap stimuli as a function of 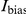 and noise intensity 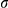. D) Oscillation frequency as a function of 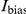 and noise intensity 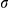.

Our simulation results are largely contrary to recordings from VN neurons performed *in vivo*. Indeed, many VN neurons accurately follow the time course of vestibular stimuli through changes in firing rate and do not display synchronization or phase locking for frequencies between 0 and 25 Hz [16]. As our modeling results described above were obtained for high values of 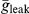 and were robust to increases in the bias current 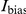, it is unlikely that the discrepancy between our model results and experimental recordings from VN neurons *in vivo* is due to a change in membrane conductance or the fact that VN neurons might be in a depolarized state *in vivo*. Thus, while our results show that increasing the bias current 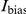 such that the firing rate increases to values seen *in vivo* did increase the range of frequencies for which our model could faithfully encode the time course of sinusoidal input, this alone was not sufficient to eliminate nonlinear synchronization for the full range of frequencies found in natural vestibular stimuli (Figures 6D,6E,7A).

Thus, we hypothesized that the increased trial-to-trial variability that is characteristic of *in vivo* conditions [25], [26] might explain this discrepancy. It is expected that such variability will limit phase locking by inducing firing at all phases of the input and thus promote the faithful encoding of the stimulus waveform by changes in firing rate (see [37] for review). We thus addressed the specific question of whether the levels of resting discharge variability displayed by VN neurons *in vivo* are sufficient to account for the suppression of nonlinear phase locking, which is observed *in vitro*, thereby allowing faithful encoding of the stimulus time course through changes in firing rate.

In order to test this hypothesis, we systematically varied both the bias current 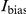 as well as the noise intensity within the experimentally observed ranges of baseline firing rates (Figure 7A) and resting discharge variability as quantified by the coefficient of variation (CV) (Figure 7B), respectively. We note that previous studies have shown that VN neurons displayed values of CV in their resting discharge ranging from 0.05 to 0.7 [16], [17] and resting discharge firing rates between 6 and 170 Hz [16], [17], [34]. Furthermore, we also explored the effects of such increased noise intensities on the models firing rate resonance, via repeated presentation of the zap stimulus for the same range of bias current values and noise intensities. For higher bias currents (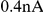) corresponding to the baseline firing rates seen under *in vivo* conditions (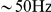), the addition of noise is seen to reduce the oscillation index (Figure 7C). Addition of noise also decreased the oscillation frequency to values near the behaviorally relevant range (Figure 7D). As an aside, we note that, for low values of bias current (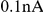), we observed a sharp increase followed by a decrease in the oscillation frequency (Figure 7D). This sharp increase at low noise intensities is consistent with previous studies showing that, for low noise, model neurons have a resonance at the spontaneous firing rate, while for higher noise intensities, the resonance frequency shifts to lower values [22]. We do not further explore this regime since VN neurons typically have baseline firing rates under *in vivo* conditions that are outside those for which this regime is observed.

**Figure 8:**
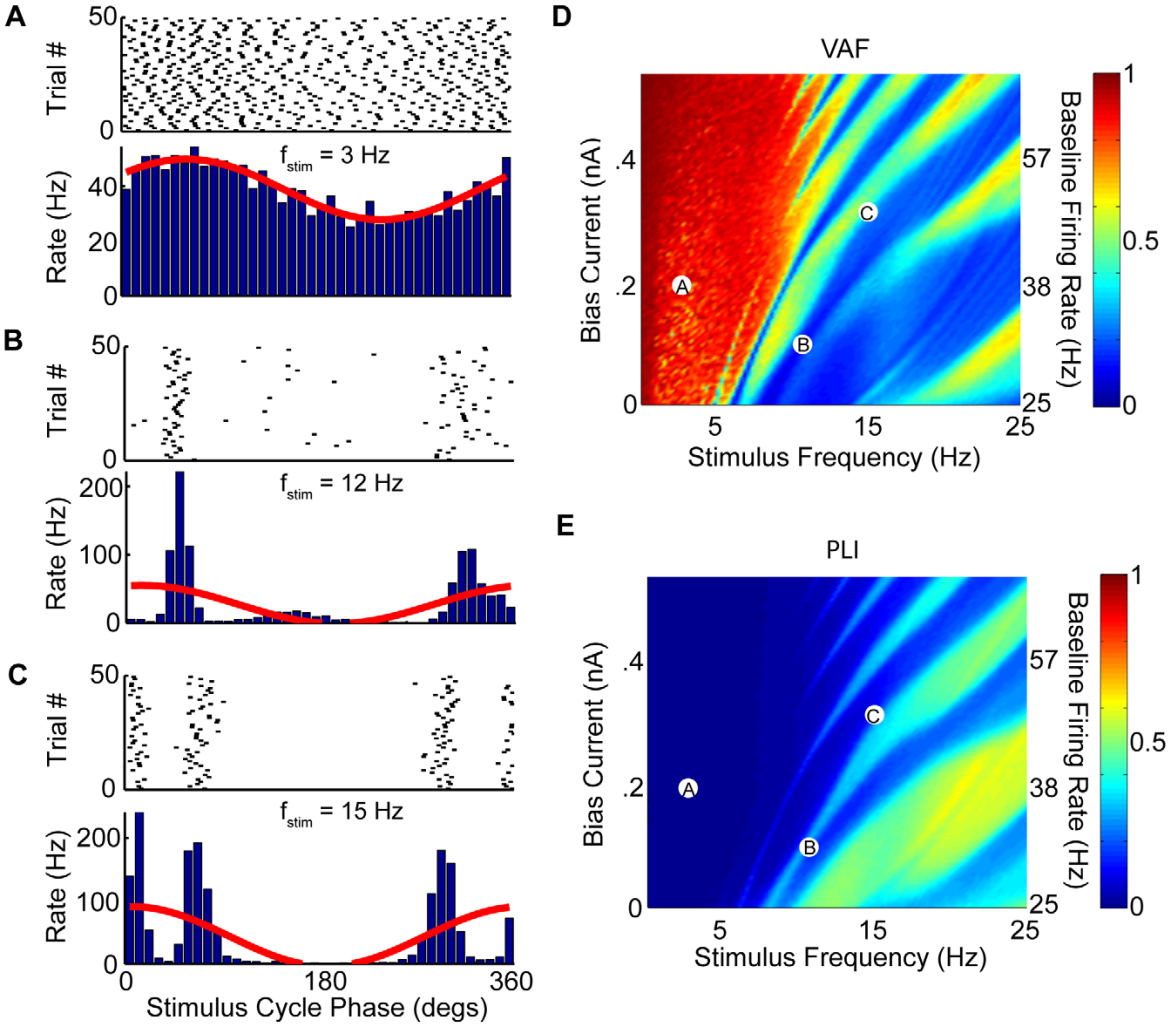
Effects of low intensity noise on synchronization to sinusoidal input and its consequences on faithful encoding of this input through changes in firing rate. We characterized the model's response to sinusoidal current injections with different frequencies using the phase histogram as before. A–C) Three example raster plots (top) and phase histograms (bottom) for the same parameter values used in Figure 6 with the best sinusoidal fits (red). D) VAF as a function of 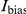 and 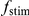. E) PLI as a function of 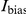 and 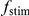. It is seen that low intensity noise somewhat disrupts phase locking but that there are still ranges of parameter values for which the model displays significant phase locking. The parameter values corresponding to panels A,B,C are also shown. Parameter values were the same as those previously described except for 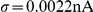.

We first recomputed phase histograms in response to sinusoidal current injection (Figures 8A,B,C) for the same range of 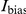 and stimulation frequencies used before but with the addition of noise with a low intensity that gave rise to low resting discharge CV values (0.04–0.24) and with bias currents giving rise to firing rates between 25–80 Hz in the absence of stimulation. We note that these overlap with the experimentally observed ranges of values [16]. We observed that this noise increased the range of stimulus phases that elicited spiking for higher stimulus frequencies, which reduced phase locking (compare Figures 8B,C with Figures 6B,C, respectively). However, this noise was not sufficient to completely eliminate phase locking as can be seen from the low VAF and high PLI values observed for high (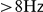) stimulation frequencies for a wide range of 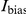 values (Figures 8D,E respectively).

**Figure 9:**
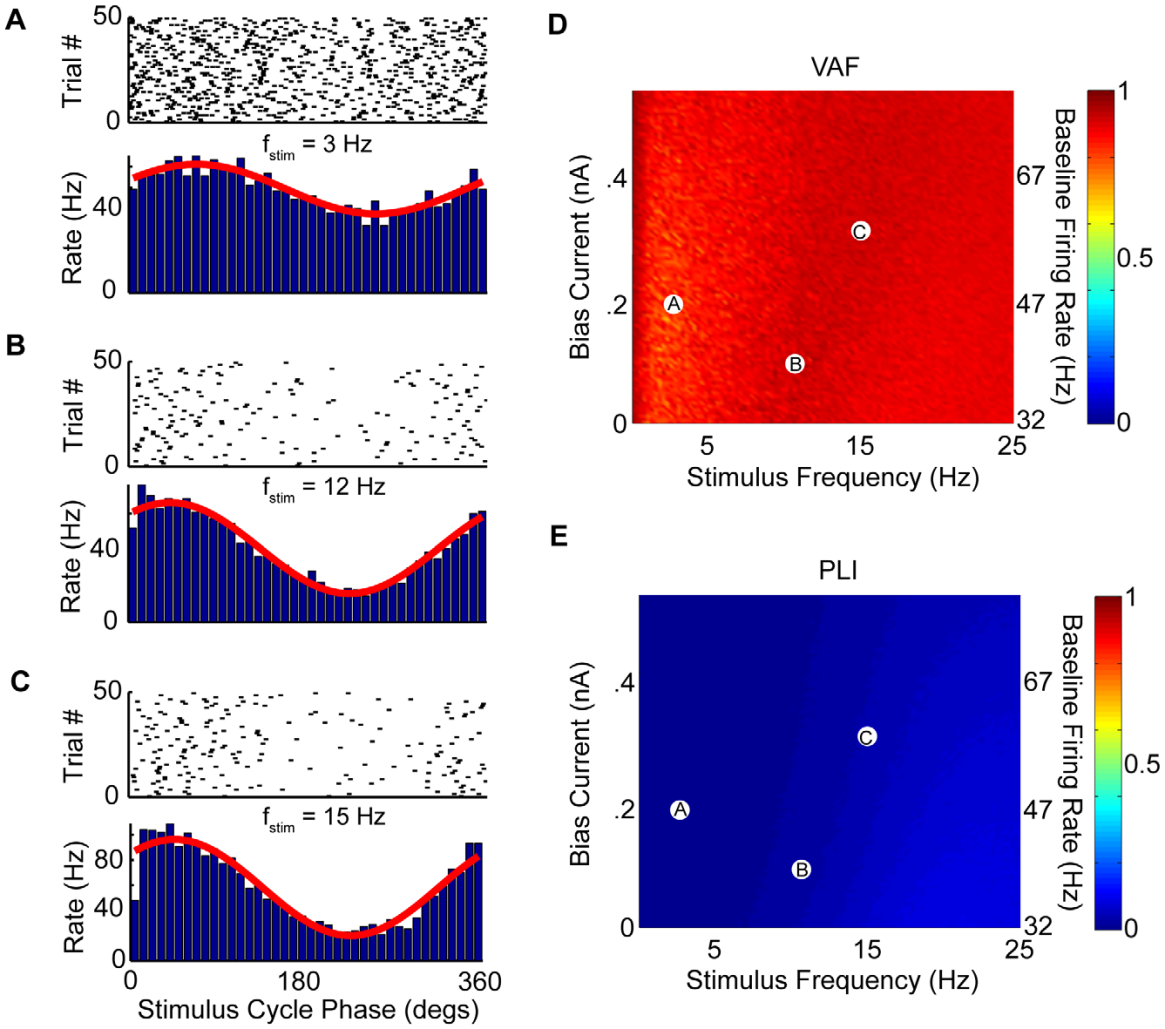
Effects of high intensity noise on synchronization to sinusoidal input and its consequences on faithful encoding of this input through changes in firing rate. We characterized the model's response to sinusoidal current injections with different frequencies using the phase histogram as before. A–C) Three example raster plots (top) and phase histograms (bottom) for the same parameter values used in Figure 8 with the best sinusoidal fits (red). D) VAF as a function of 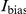 and 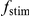. E) PLI as a function of 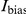 and 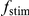. It is seen that high intensity noise eliminates phase locking and promotes faithful encoding of the input waveform by changes in firing rate as can be seen from the sinusoidal phase histograms, high VAF values, and negligible PLI values. The parameter values corresponding to panels A,B,C are also shown. Parameter values were the same as those previously described except for 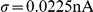.

We next performed simulations with a higher noise intensity giving rise to higher resting discharge CV values (0.5–0.7) and bias current giving rise to firing rates from 35–85 Hz. Our results show that the phase histograms in response to sinusoidal current injection were all sinusoidal in shape, even for parameters that gave rise to phase locking in the absence of noise (compare Figures 9A,B,C with Figures 6A,B,C, respectively). This indicates a lack of phase locking as every phase of the input can now elicit spiking. We recomputed the VAF as a function of 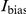 and stimulus frequency and found large (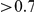) values over the entire range explored (Figure 9D). Consequently, the model displayed negligible phase locking as quantified by the PLI (Figure 9E). Note that the range of values of VAF and PLI used in Figures 9D and 9E, respectively, were the same as those used previously (compare Figures 9D,E with Figures 6D,E and Figures 8D,E, respectively). As such, this noise intensity was sufficient to eliminate nonlinear phase locking and thereby give rise to faithful encoding of the stimulus waveform through changes in firing rate for all stimulus frequencies within the behaviorally relevant range.

In order to verify the robustness of our results, we also computed a second measure of nonlinear synchronization, the nonlinearity index (NI, see Methods), that is based on the ratio of the Fourier coefficient amplitude squared at the second harmonic to that at the stimulus frequency. This measure had qualitatively similar behavior to that of the PLI measure as a function of the bias current 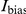, stimulus frequency, and noise intensity (compare Figure S3 to Figures 6,8,9).

Finally, in order to test that these results were not an artifact of our using current input, we used conductance input rather than current input stimuli in our model. The effect of noise on phase locking in this model (Figure S4) were in qualitative agreement with those shown in Figures 6,8, and 9, illustrating the robustness of our main result to the type of input used. We note that this outcome was expected given that increasing the membrane conductance alone was not sufficient to completely eliminate phase locking over the behaviorally relevant frequency range.

**Figure 10:**
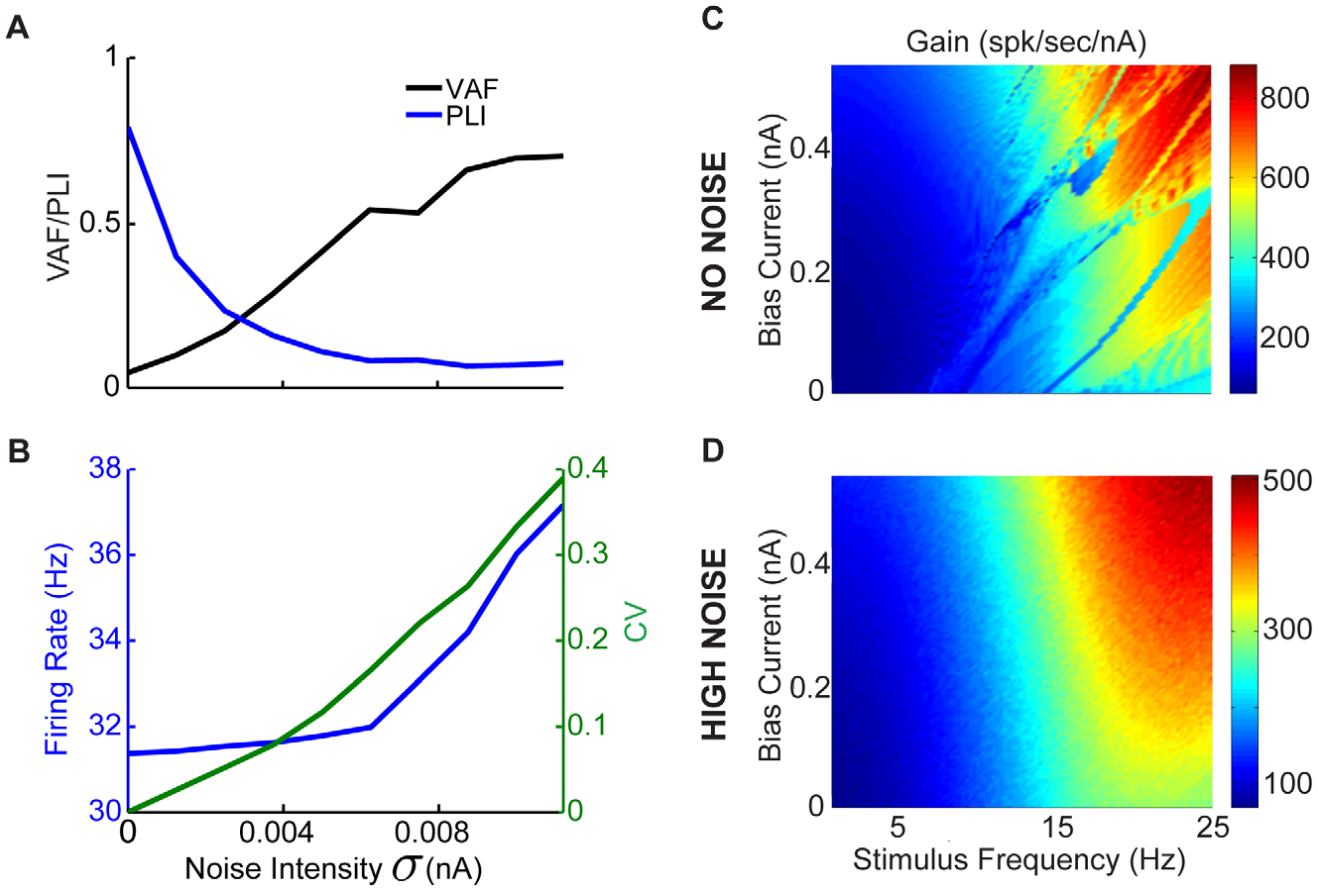
Effects of varying noise intensity on the VAF and PLI. A) Increasing noise intensity results in decreased PLI and consequently increased VAF values for 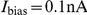 and 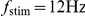. B) Increasing noise intensity also results in increased resting (ie 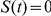) discharge rate as well as increased spiking variability as quantified by CV. C) Gain obtained from our model with no noise (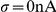) as a function of 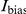 and 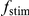. D) Gain obtained from our model with high noise intensity (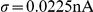) as a function of 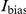 and 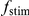. It is seen that for a given value of 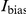 the gain increases as a function of the input frequency 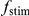 until about 22 Hz.

The effects of noise intensity on our models ability to accurately encode the time course of sinusoidal current injections through changes in firing rate are summarized in Figure 10. While the PLI rapidly decreases as a function of increasing noise intensity, the VAF rapidly increases (Figure 10A). For comparison, the resulting firing rate and CV values in the absence of stimulation are also shown for the same noise intensities (Figure 10B). Because high noise intensities were sufficient to eliminate nonlinear phase locking from our model, we used linear systems analysis to characterize the relationship between input and output in our model. Specifically, we computed the gain (i.e. the coefficient relating input and output) as a function of 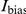 and stimulus frequency. Our results show that the gain increases smoothly as a function of stimulation frequency for a given value of 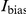 in the presence of high noise but not so when noise is not present (Figures 10C, D). This result is important as previous studies conducted *in vivo* have shown that VN neurons generally display increasing gains as a function of stimulus frequency [16], [17]. Our results therefore suggest that the high-pass filtering characteristics seen in most VN neurons *in vivo* which are due, at least in part, to an intrinsic resonance. This resonance is attenuated by the high resting discharge variability that results from the intense convergent synaptic input that the cell receives under *in vivo* conditions.

## Discussion

### Summary of results

The goal of this study was to resolve an apparent discrepancy between the responses of VN neurons to current injection *in vitro* and to sensory input *in vivo*. VN neurons are prone to display nonlinear responses such as synchronization to the peaks of sinusoidal current injection *in vitro*. In contrast, studies performed *in vivo* have shown that VN neuron can respond to sensory input through changes in firing rate that accurately follow variations in sensory stimulation over a wide frequency range [16]. We investigated the cause for this discrepancy by subjecting a mathematical model based on the Hodgkin-Huxley formalism of *in vitro* VN neuron activity to *in vivo* conditions.

Our results show that this model displays membrane potential oscillations that persisted for a wide range of parameter values. These oscillations give rise to a resonance in the membrane potential which is transmitted to the spike train, causing nonlinear behavior such as synchronization or phase locking over the natural stimulus frequency range (0–20 Hz). It is well known that neural variability resulting from the intense synaptic bombardment to which VN neurons are subjected to *in vivo* will promote faithful encoding of the stimulus waveform through changes in firing rate [37]. As such, we tested the hypothesis that the levels of resting discharge variability seen under *in vivo* conditions could account for the fact that some VN neuron classes do not display synchronization *in vivo*. To do so, we added noise whose intensity was calibrated in order to match the resting discharge variability experimentally observed in VN neurons under *in vivo* conditions. We found that low noise intensities did not completely eliminate phase locking behavior. In contrast, we found that high noise intensities almost completely eliminated phase locking and that our model could now faithfully encode the time course of sinusoidal current injections at frequencies contained within 0–20 Hz for a wide range of input bias currents. These results are consistent with experimental recordings from VN neurons *in vivo*, suggesting that the addition of noise in the *in vivo* condition underlies the discrepancy between the responses of VN neurons to current injection *in vitro* and to sensory input *in vivo*. Furthermore, they suggest that the vestibular system uses increases in variability to increase the fidelity of encoding by single neurons. This strategy appears to be found across several sensory systems (reviewed in [37]).

### Correspondence between anatomy and function in VN

In the present study, we focused on the type B neurons as observed *in vitro*. This is because these neurons display the greatest tendency to respond to sinusoidal current injection with synchronization as well as spike frequency adaptation. In contrast, type A neurons show a sustained tonic response and faithfully follow the time course of sinusoidal current injections that are up to three times larger than those followed by type B neurons [8]–[11], [13]. The differences between type A and type B neurons are thought to be mediated by differences in the levels of different membrane conductances [12], [14]. In particular, type B neurons display larger calcium-activated conductances [13]. Such currents mediate spike frequency adaptation (see [38], [39] for review). Theoretical studies have shown that spike frequency adaptation leads to high-pass filtering of time varying stimuli [40]–[42], which is consistent with our modeling results showing an increased gain for higher frequencies. We note that one could use the same model as was used here in order to mimic the activity of type A VN neurons by changing membrane conductances as was done previously [14]. We predict that a model of type A VN neuron activity would not display phase locking for the sinusoidal current injections considered here but would display phase locking for larger amplitudes.

*In vivo* studies have found three major functional neuronal classes in MVN that are based on the responses to voluntary eye movements and passive whole-body rotation: 1) Vestibular-Only (VO) neurons, 2) Position-Vestibular-Pause (PVP) neurons, 3) Floccular Target neurons (FTN). VO neurons project to the spinal cord and are thought to mediate vestibulo-spinal reflexes that control posture [43]–[45], as well as cerebellum and thalamus [46], [47], where they are thought to play a role in spatial orientation computation. The vestibular system also generates the vestibulo-ocular reflex (VOR) that functions to effectively stabilize gaze by moving the eye in the opposite direction to the on-going head motion. The three-neuron arcs mediating the VOR are well characterized. The primary pathway consists of projections from afferents to PVP neurons, which in turn project to extraocular motoneurons that control the eye muscles. A secondary pathway is mediated via FTN neurons that receive direct input from the Floccular lobe of the vestibular cerebellum and also project to the extraocular motoneurons. The correspondence between type A and B MVN neurons as observed *in vitro* and the different functional classes observed *in vivo* is not well understood in general. The most direct link that has been made to date is based on the findings of electrophysiological and anatomical studies that suggest a subpopulation of type B neurons receive input from Floccular purkinje cells, such that they most likely correspond to the FTN neurons which have been characterized *in vivo* [20], [21]. This correspondence between type B cells and FTN cells, however, is unexpected since *in vivo* experiments have shown that FTN neurons do not display robust phase locking and instead respond to sinusoidal head rotations through changes in firing rate that scale with stimulus intensity for frequencies spanning the behaviorally relevant range *in vivo* [16]. Thus, our results provide a potential explanation of this discrepancy originating in the intense synaptic bombardment that these neurons receive *in vivo*.

The correspondence between VO and PVP neurons *in vivo* and type A/B neurons *in vitro* is not known. However, previous studies have shown that PVP neurons display nonlinear phase locking behavior in response to high frequency (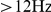) sinusoidal rotations [16]. This is consistent with our modeling results showing that phase locking is not abolished for low noise intensities (Figure 8). Our results therefore predict that: i) PVP neurons should have type B like responses *in vitro*; ii) PVP neurons with low resting discharge rates will display a greater tendency for phase locking and, iii): this tendency is a consequence of their low resting discharge variability. Previous studies have reported that VO neurons do not display phase locking dynamics but have only explored frequencies between 0 and 4 Hz [48]. Further studies are needed to explore VO neuron responses to higher stimulus frequencies and might help elucidate their correspondence with either type A or type B neurons.

In conclusion, while it is clear that the filtering properties of VN neurons as observed *in vivo* are shaped by intrinsic mechanisms [13], our simulations are consistent with a growing body of literature emphasizing the role of network mechanisms [42], [49] such as synaptic bombardment that is present under *in vivo* conditions affecting their responses to sensory input.

### Sources of variability in VN

What are the sources of resting discharge variability in VN neurons? A unique aspect of the vestibular system, compared to other sensory systems, is that information processing is strongly multisensory and multimodal at the first stage of central processing. This occurs because the vestibular nuclei receive inputs from a wide range of cortical, cerebellar, and other brainstem structures in addition to direct inputs from the vestibular afferents. First, there is complete overlap in the terminal fields of regular and irregular afferents in each of the major subdivisions of the vestibular nuclei [50], and the results of electrophysiological studies have shown that about half of the VN neuron population receive significant input from both afferent classes [5], [6]. Additionally, not only do neurons typically receive convergent input from otolith as well as canals afferents, but there is an impressive convergence of extra-vestibular information within the VN (reviewed in [51]). Notably, sensory inputs encoding somatosensory, proprioceptive, and visual information as well as premotor signals related to the generation of eye and head movements are sent directly to the vestibular nuclei. In alert animals, these extra-vestibular signals strongly modify the processing of vestibular information during our everyday activities, such that this convergence plays an important role in shaping the simple sensory-motor transformations that mediate vestibulo-ocular and vestibulo-spinal reflexes as well as higher-order vestibular functions, such as self-motion perception and spatial orientation. Thus, as a result of their cortical, cerebellar, and brainstem and afferent input afferents, VN neurons are likely to receive substantial synaptic bombardment *in vivo*. For example, extracellular recordings in the cerebellar flocculus reveal irregularities in the spontaneous simple spikes firing rate of the output neurons (i.e. Purkinje cell) [52]. This provides a clear source of variability to FTN neurons which might explain their lack of synchronization to sensory stimulation as predicted from our modeling results.

### Differences between *in vivo* and *in vitro* conditions in VN neuronal activity

Previous reports have found that the high conductance state of neurons *in vivo* can have a significant influence on their processing of synaptic input through changes in intrinsic dynamics [27], [53]–[55]. Specifically, these changes consist of: 1) increased synaptic input that is dominated by excitation that acts as a net depolarizing bias; 2) increased membrane conductance and; 3) increased variability. In general, bridging the gap between *in vivo* and *in vitro* conditions is not well understood because it is not clear which combination the three aforementioned effects is responsible for the observed changes in dynamics. For example, both changes in the depolarization bias as well as in variability can alter burst dynamics in thalamocortical neurons [54], [56].

Previous studies have investigated the effects of *in vivo* conditions on the activity of VN neurons [14], [57], [58]. In particular, it has been proposed that heterogeneities might allow for the VN neuron population to accurately encode the time course of vestibular stimuli while maintaining nonlinear synchronization at the single neuron level [58]. This hypothesis is contrary to more recent experimental results showing that many neurons in the VN, such as FTNs, do not display phase locking *in vivo* [16]. Our results instead predict that increased variability seen under *in vivo* conditions can account for the fact that these neurons accurately follow the time course of vestibular stimuli through changes in their firing rates and that nonlinear behavior such as phase locking occurs because of intrinsic rather than network dynamics.

Moreover, it has been proposed that *in vivo* conditions could be mimicked in VN neurons by increasing the bias current, thereby increasing the firing rate [14], [57]. Our results show that increases in both bias current and membrane conductance are not sufficient to eliminate synchronization for the parameter values used in our model. Instead, our results predict that variability in the form of noise is the main reason for many VN neurons not displaying synchronization *in vivo*. The mechanism by which this noise attenuates synchronization is not by increasing the baseline firing rate but instead by enabling the firing of action potentials at all phases of the stimulus cycle. This prediction can be tested experimentally *in vitro* by mimicking *in vivo* conditions through the dynamic clamp technique [25]. Similar variability-related effects have been observed experimentally in recordings from entorhinal cortical stellate cells *in vitro* [27]. Indeed, these cells show a strong tendency to display subthreshold membrane potential oscillations in the theta range *in vitro* [59] but no significant peak in the theta range has been observed in their activities in awake behaving animals [60]. This suggests that these subthreshold membrane oscillations are strongly attenuated *in vivo*. The results of Fernandez and White [27] support this viewpoint as they observed weaker oscillations when they increased conductance and variability through dynamic clamp *in vitro*.

In particular, we note that our model did not include the inward rectifier current 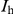 that is known to be present in VN neurons [21]. While this current has been previously shown to increase the magnitude of membrane potential oscillations [23], it is unlikely to be activated in the depolarized state characteristic of *in vivo* conditions in VN neurons [21]. Indeed, in order to activate 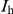, the membrane potential must be brought to about 15 mV below the spiking threshold for at least 300 ms [21]. Such a large hyperpolarization leads to a cessation of firing as observed *in vitro* that lasts for at least 300 ms. However, VN neurons are spontaneously active with firing rates of 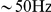 in vivo and do not respond to vestibular stimuli (for the intensities typically used in vivo studies) with a complete cessation of firing that lasts 300 ms [17]. Instead, VN neurons smoothly encode variations in head velocity through changes in their firing rate but their firing rates does not reach zero. Thus, it seems unlikely that the membrane potential would reach the values that are necessary to activate 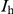.

Finally, we note that there exist highly detailed compartmental models of VN neurons that are more morphologically realistic than the model used here [61]. While it would be more realistic to use a detailed compartmental model with an anatomically accurate dendritic tree, such a model would have a significantly greater number of parameters than our current one. Justifying the values used for many of these parameters (i.e. the precise location, strength, and dynamics of afferent synapses on the dendritic tree) would be non-trivial at best. Based on our results, we can conclude that taking into account the shape of the dendritic tree of VN neurons is not necessary to explain the discrepancy between *in vitro* and *in vivo* results. Nevertheless, future experiments should focus on understanding the effects of dendritic processing in VN neurons.

### Stochastic resonance in VN neurons promotes linear coding: functional consequences

Our results have demonstrated that noise can enhance signal transmission in our model VN neuron. Such enhancement of signal transmission by noise is often referred to as stochastic resonance [62]–[67], a phenomenon by which noise enhances the transmission of a subthreshold signal (i.e. a signal whose intensity is not sufficient to induce spiking activity on its own). We note that our result is, strictly speaking, not stochastic resonance since we chose model parameter values within the suprathreshold regime (i.e. the stimulus could induce action potential firing in the absence of noise). However, in our model, one of the effects of the noise is to induce firing for subthreshold stimulus values. Such effects have been widely discussed before and are commonly referred to as the Òlinearization of systems by noise [37], [68].

While this linearization by noise enables our model VN neuron to faithfully encode the time course of input within the natural frequency range (0–20 Hz), such encoding will only be seen for a finite range of stimulus amplitudes. Indeed, stimuli with larger amplitudes are expected to elicit nonlinear synchronization in VN neurons despite high trial-to-trial variability. In particular, such large amplitude stimuli might lead to activation of 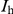 from the argument above. The putative function of such nonlinear encoding remains a mystery and should be the focus of future studies.

What is the functional role of suppressing synchronization in VN neurons *in vivo*? It is clear that such synchronization in the form of phase locking is used extensively in the auditory system [69]–[76]. Previous studies have shown that the addition of noise leads to a linearization of the steady state current-response relationship (i.e. the f-I curve) in model neurons [68]. Such linearization of the f-I has also been shown to give rise to gain control mechanisms [77]–[79] which will extend the dynamic range (i.e. the range of input values that can be coded through a change in output) of a neuron. We propose that increased variability serves to increase the dynamic range of VN neurons and therefore promote more faithful encoding of the stimulus time course through changes in firing rate over a wider range of vestibular stimulus intensities encountered by the organism in the natural environment. This prediction can be tested *in vitro* using the aforementioned dynamic clamp technique.

## Methods

### Model

We used a conductance based Hodgkin-Huxley-type model of VN neuron activity *in vitro* [14], [80], [81]. The model includes spiking sodium, persistent sodium, delayed rectifier potassium, calcium, and calcium-activated potassium currents. We note that our model did not include the hyperpolarization activated inward rectifier current 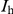 which is present in VN neurons [21] and that addition of this current did not qualitatively affect the nature of our results (data not shown). The model is described by the following system of stochastic differential equations:
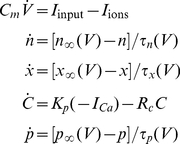
(1)where 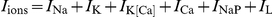 are the ionic currents, which are given by
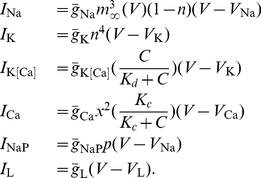
(2)


The dynamical variables are the membrane voltage 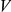, the calcium concentration 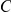, and the activation variables 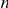, 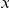, and 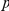. Although synaptic inputs are most accurately described by fluctuating conductances as described by Destexhe et al. [26], an effective synaptic input [22] can be modeled as an additive current decomposed into three components: a bias current, additive current fluctuations, and a stimulus modulation current. As such, we had 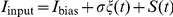 where 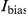 is the bias current and 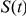 is the stimulus current injection. 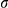 is the noise intensity and 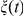 is low pass filtered (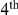-order Butterworth with 50 Hz cutoff) [82] Gaussian white noise with mean zero and standard deviation unity. The activation variables 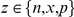 obey the following equation:
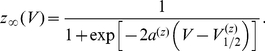
(3)Furthermore, while the time constants 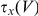 and 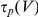 are taken to be independent of the membrane voltage V, the voltage dependent time constant 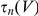 is given by
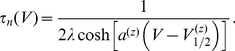
(4)Unless otherwise indicated, parameter values were taken as originally tuned [14], and are listed as follows: 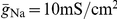, 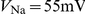, 
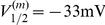
, 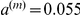, 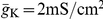, 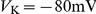, 
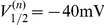
, 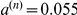, 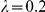, 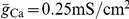, 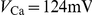, 
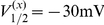
, 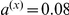, 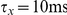, 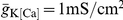, 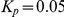, 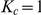, 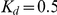, 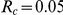, 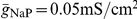, 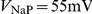, 
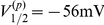
, 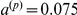, 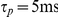, 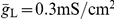, 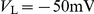, and 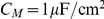. The model equations were integrated numerically using an Euler-Maruyama numerical integration technique [83] with an integration time step of 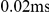.

If the time scale at which 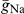 and 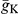 vary at is much smaller than all other time scales in the model, then one can replace the sodium and potassium currents in equation (1) by their average values during an action potential. This is the case for the parameter values used here. Indeed, the time constant of 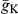 is 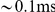 while the minimum time constant of all other processes in our model is 5 ms (note that 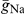 tracks the membrane potential instantaneously and thus has an effective time constant of zero). We note that, for our parameter values, the average value of summed sodium and potassium currents during an action potential is 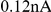, which is an order of magnitude less than the range of bias currents used in this study. As such, our approach of setting 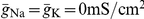 is valid if one is interested in looking at the dependence of these oscillations on parameter values.

Neurons are known to receive massive amounts of synaptic bombardment from afferent inputs *in vivo*, which puts them into a high-conductance state. Such conditions are characterized by a depolarized and fluctuating membrane potential with a reduction in input resistance (or equivalently an increase in membrane conductance) [26]. Although each individual synaptic input can be accurately modeled by including the presynaptic action potential sequence, the increased membrane conductance and membrane potential fluctuations due to synaptic bombardment onto a neuron can be accurately reproduced by increasing the leak conductance, adding a depolarizing bias current, and adding a noisy current [22], [82], [84]. We note that increasing the leak conductance in order to mimic the increased membrane conductance due to synaptic bombardment is used in dynamic clamp experiments [27].

In order to verify the robustness of our results to more biophysical conditions, we also modeled our sinusoidal stimulus input using an excitatory conductance-based input rather than a simple current input. In this case we used an input current 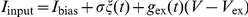, with the excitatory reversal potential 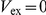. The excitatory conductance was set to 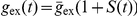, where 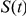 is now a sinusoid with amplitude of unity, ensuring that 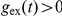. The overall strength of the sinusoidal input is then set by 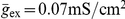, the value of which was chosen to achieve a comparable firing rate modulation as achieved for equivalent simulations with current input.

### Measures

For membrane potential responses to step current inputs, the oscillation index is calculated from the response in the time domain V(t), from the following equation:
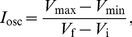
(5)where 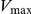 is the maximum voltage occurring after the input step onset, and 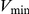 is the minimum voltage that occurs after the maximum. 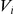 and 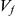 denote the initial and final values of the voltage, respectively.

In the case of zap current injection, the oscillation index was computed from the envelope of the amplitude modulated membrane voltage response. The envelope was computed by subdividing the membrane potential waveform into windows of length 100 ms and by taking the maximum value within each window. The resulting curve was then low-pass filtered (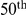-order low-pass FIR filter with 1.875 Hz cutoff). The oscillation index is then given by the envelope maximum minus the value at t = 0. For the spiking activity, the oscillation index is computed in a manner similar to that described above but using the instantaneous firing rate (i.e. the reciprocal of the ISIs) waveform. In that case, each window was 400 ms long and the filter was a 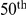-order low-pass FIR filter with 0.625 Hz cutoff.

We also characterized the model's response to sinusoidal current injections that spanned the behaviorally relevant frequency range (0–20 Hz). As done before [14], to convert current density to current, we assume that our model neuron is spherical with a radius of 20 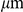, so that 10 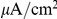 is equivalent to 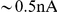. This was done in order to facilitate the comparison of our simulation with experimental data. We used sinusoid amplitudes of 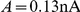, as were previously used experimentally *in vitro* [13]. Sinusoidal current injections of a given frequency lasting one cycle were repeatedly presented with the model neuron's initial conditions randomized before each presentation, until 100 seconds of data had been generated for each combination of 100 stimulus frequencies and 100 values of bias current. A cycle histogram was then computed and normalized in order to give the firing rate R(t), as a function of the stimulus phase. The firing rate was then fit to the optimal linear regression model defined as 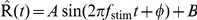, as is done experimentally [18], [19], [85]. Although fitting the phase 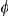 of 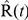 is nonlinear, an optimal linear fit was made for many possible phase values held constant, and the best linear fit taken. The goodness of the fit is then quantified by the variance-accounted-for (VAF) given by the following equation:
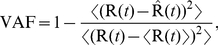
(6)where 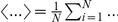 with 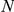 the number of bins. In the case of a perfect fit, the numerator is equal to zero and the VAF is equal to its maximum value of one. The worst possible fit results in a the minimal VAF of zero. The gain and phase of the response are then calculated as the amplitude of the fit sinusoid normalized by the amplitude of the stimulus and the phase shift of the fit with respect to that of the stimulus, respectively [18], [19], [85].

The phase locking index (PLI) is computed using the entropy of the cycle histogram [86]. Unlike measures of vector strength [87], this measure can quantify the degree of phase locking present in multi-peaked phase histograms, as present in our case. It is given by:
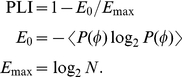
(7)where 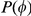 is the probability of firing a spike as a function of stimulus phase. 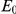 gives the entropy of the probability distribution and 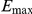 is the maximum entropy possible and is that of a uniform distribution. The PLI thus ranges between 0 and 1. As phase locking is a nonlinear phenomenon, we supplement this measure with the use of an additional more intuitive measure we refer to as a nonlinearity index (NI). This is done by taking the Fourier transform of the of the firing rate, 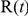, in response to sinusoidal stimulation. We then take the ratio of the magnitudes of the Fourier coefficient squared (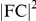) at three times the stimulus frequency divided by that at the stimulus frequency. We thus define NI as:
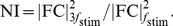
(8)If the firing rate is a linear function of the sinusoidal stimulus, then it can only contain power at the stimulus' frequency. If there is phase locking, however, then the magnitude squared of the Fourier coefficients at higher harmonics of the stimulus frequency will be non-zero.

### Linearized model

In the subthreshold regime with spiking sodium and rectifying potassium conductances set to zero, our nonlinear neuron model reduces to the following:
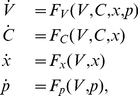
(9)where 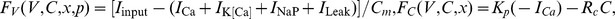 and 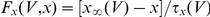, and 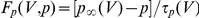. The steady state values of all dynamical variables, 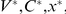 and 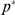, can then be found numerically by solving the four equations 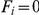, for 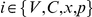. The system can then be linearized in the neighbourhood of these fixed points by Taylor expanding the four functions 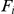 and keeping only first order terms in the expansions [88]. Redefining the four system variables in terms of their deviation from steady state, 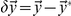 with the vector of dynamical variables defined as 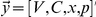, 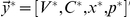, and 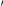 denotes vector transposition, the linearized system can then be described by the system of equations:
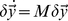
(10)where 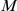 is the Jacobian, which is given by:
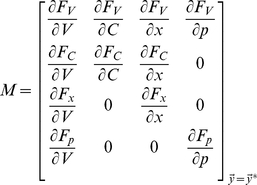
(11)


Finally, the Matlab function eigs is used to find the four eigenvalues, 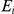 for 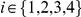, of the matrix 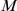 ordered by their magnitudes. All four eigengalues have a negative real part implying that the fixed point is stable. However, the last two eigenvalues were complex conjugates of each other, which implies the existence of oscillatory dynamics in the time course of the perturbations as they decay to zero. The frequency of such oscillations is given by the imaginary part of the third or fourth eigenvalues divided by 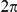. In order to assess the strength of these oscillations, the linearized model was simulated for step current inputs and the same oscillation index previously used for step current responses was calculated.

## Supporting Information

Figure S1
**Effects of increased persistent sodium conductance on membrane potential oscillations.** The model's membrane potential response to step current input was characterized for physiologically plausible ranges of bias current and persistent sodium conductance values. A–C) Example responses and the normalized squared magnitude of their Fourier transforms. These correspond to parameter values as follows: A) 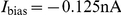, 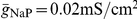, B) 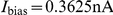, 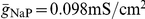, and C) 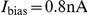, 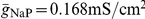. D) Oscillation index as a function of 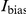 and 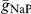. E) Oscillation frequency as a function of 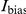 and 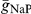. The parameter values corresponding to panels A,B,C are also shown. Other parameter values were 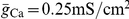, 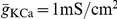, 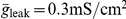, and 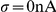.(TIF)

Figure S2
**The linearized models response to step input agrees quantitatively with that of the full model.** A) (left) Oscillation index and (right) oscillation frequency as a function of 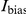 and 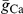 for the linearized model. B) (left) Oscillation index and (right) oscillation frequency as a function of 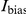 and 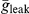 for the linearized model. C) (left) Oscillation index and (right) oscillation frequency as a function of 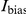 and 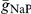 for the linearized model. In each case, other parameter values were the same as those used for the full model shown in Figures 2,3, and S1, respectively.(TIF)

Figure S3
**A nonlinearity index (NI) gives qualitatively similar results to those obtained with the PLI measure.** A) NI as a function of the bias current 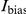 and stimulus frequency without noise. B) Example PSTH responses corresponding to 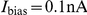 and 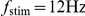. C) The squared magnitude of the Fourier transform of the PSTH response. D) NI as a function of the bias current 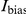 and stimulus frequency with low intensity noise. E) Example PSTH responses corresponding to 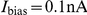 and 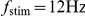. F) The squared magnitude of the Fourier transform of the PSTH response. G) NI as a function of the bias current 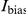 and stimulus frequency with high intensity noise. H) Example PSTH responses corresponding to 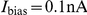 and 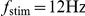. I) The squared magnitude of the Fourier transform of the PSTH response.(TIF)

Figure S4
**Synchronization to sinusoidal conductance input and the effects of noise.** A) VAF as a function of the bias current 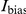 and stimulus frequency without noise. B) PLI as a function of the bias current 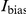 and stimulus frequency without noise. C) NI as a function of the bias current 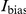 and stimulus frequency without noise. D) VAF as a function of the bias current 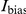 and stimulus frequency with low intensity noise. E) PLI as a function of the bias current 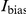 and stimulus frequency with low intensity noise. F) NI as a function of the bias current 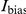 and stimulus frequency with low intensity noise. G) VAF as a function of the bias current 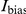 and stimulus frequency with high intensity noise. H) PLI as a function of the bias current 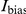 and stimulus frequency with high noise. I) NI as a function of the bias current 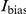 and stimulus frequency with high intensity noise. All other parameters values were the same as those used in the equivalent simulations shown in Figures 6, 8, and 9 for current input, with the additional excitatory synaptic conductance 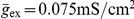.(TIF)
