# Supplementary figures and images for: In vivo Conditions Induce Faithful Encoding of Stimuli by Reducing Nonlinear Synchronization in Vestibular Sensory Neurons
Source: PLoS Comput Biol. 2011 Jul 21;7(7):e1002120. doi: 10.1371/journal.pcbi.1002120 (PMC3140969; doi:10.1371/journal.pcbi.1002120)

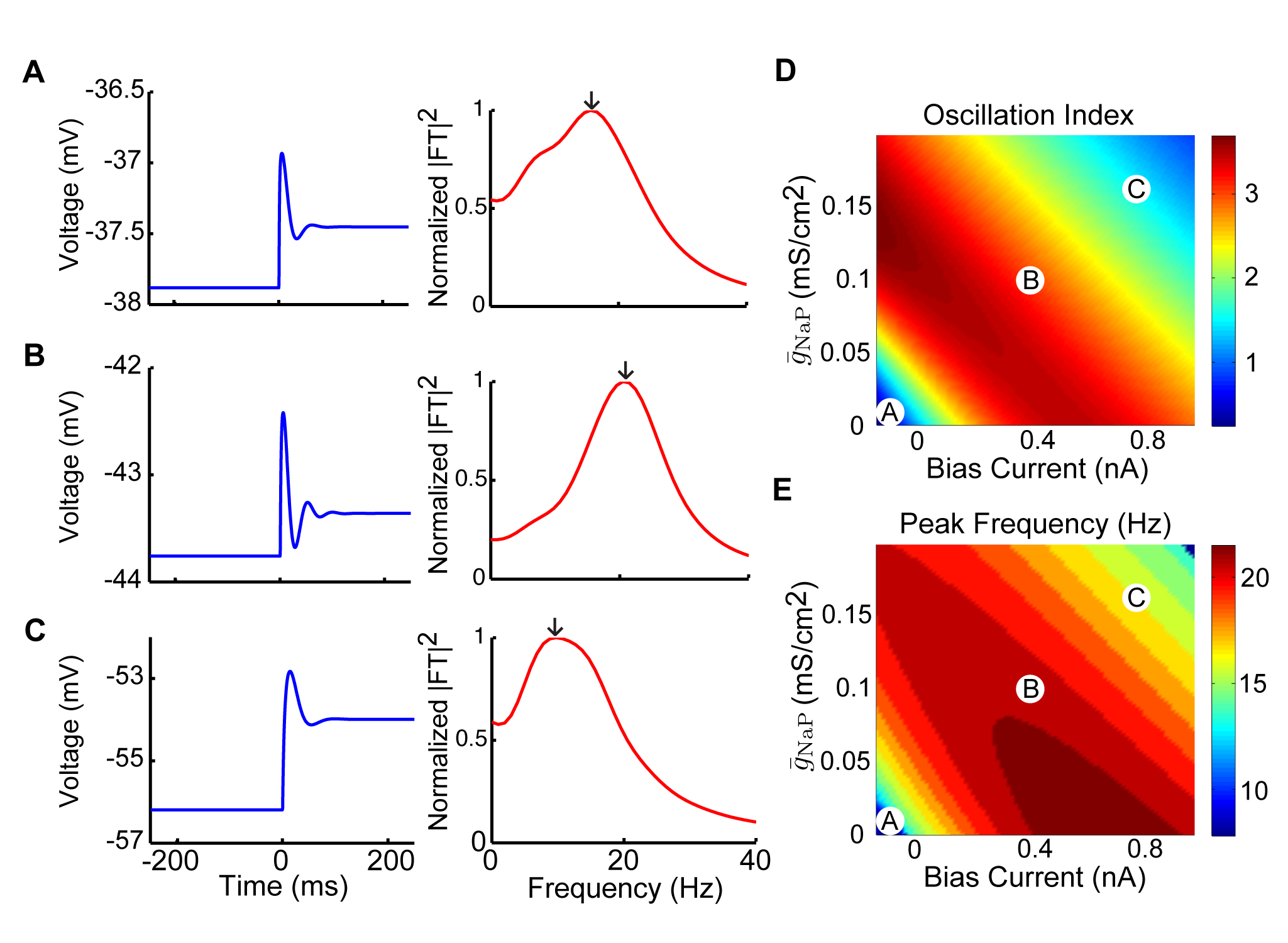

Supplement: Figure S1 — Effects of increased persistent sodium conductance on membrane potential oscillations. The model's membrane potential response to step current input was characterized for physiologically plausible ranges of bias current and persistent sodium conductance values. A–C) Example responses and the normalized squared magnitude of their Fourier transforms. These correspond to parameter values as follows: A) , , B) , , and C) , . D) Oscillation index as a function of and . E) Oscillation frequency as a function of and . The parameter values corresponding to panels A,B,C are also shown. Other parameter values were , , , and . (TIF) [file pcbi.1002120.s001.tif]

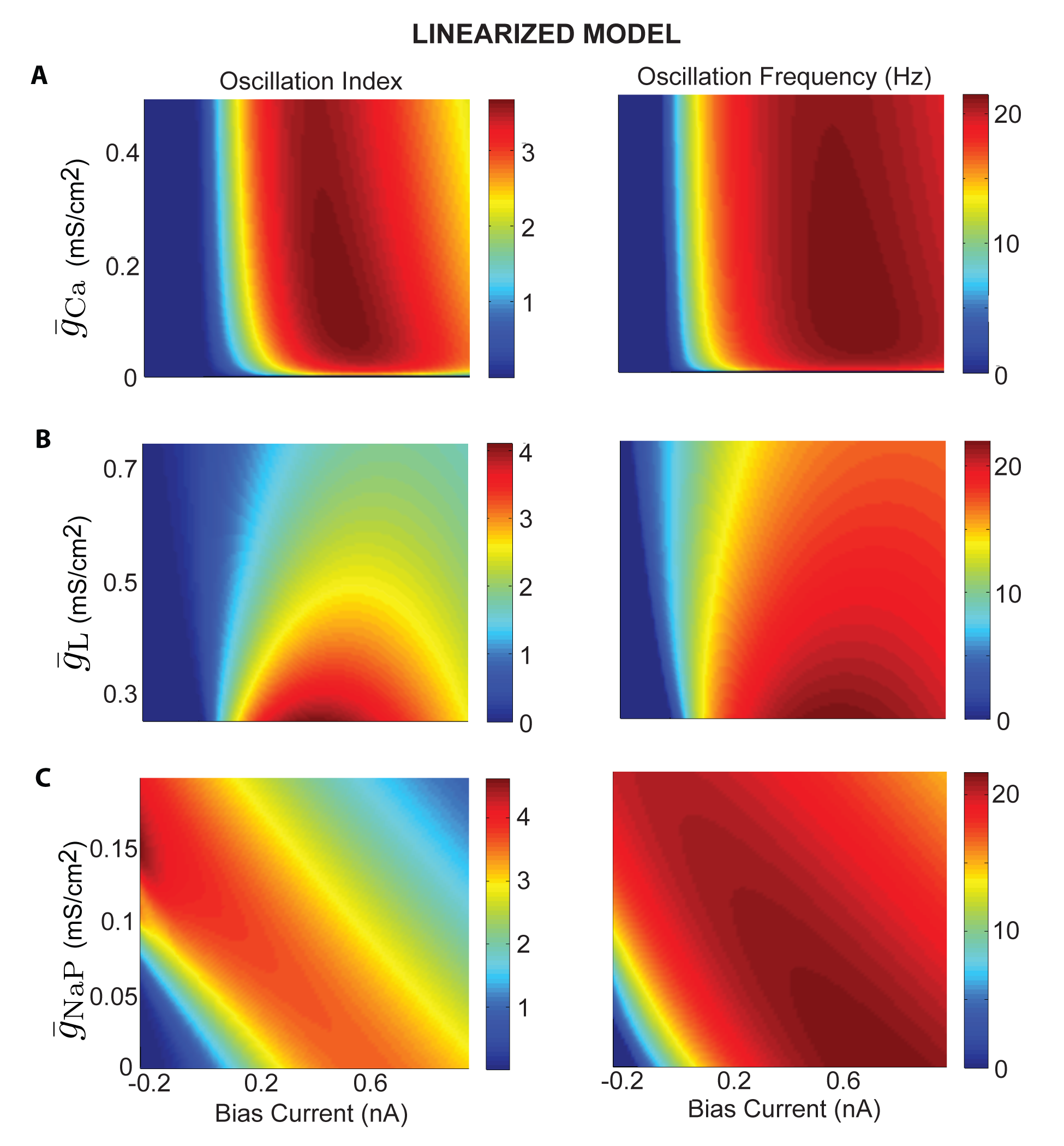

Supplement: Figure S2 — The linearized models response to step input agrees quantitatively with that of the full model. A) (left) Oscillation index and (right) oscillation frequency as a function of and for the linearized model. B) (left) Oscillation index and (right) oscillation frequency as a function of and for the linearized model. C) (left) Oscillation index and (right) oscillation frequency as a function of and for the linearized model. In each case, other parameter values were the same as those used for the full model shown in Figures 2,3, and S1, respectively. (TIF) [file pcbi.1002120.s002.tif]

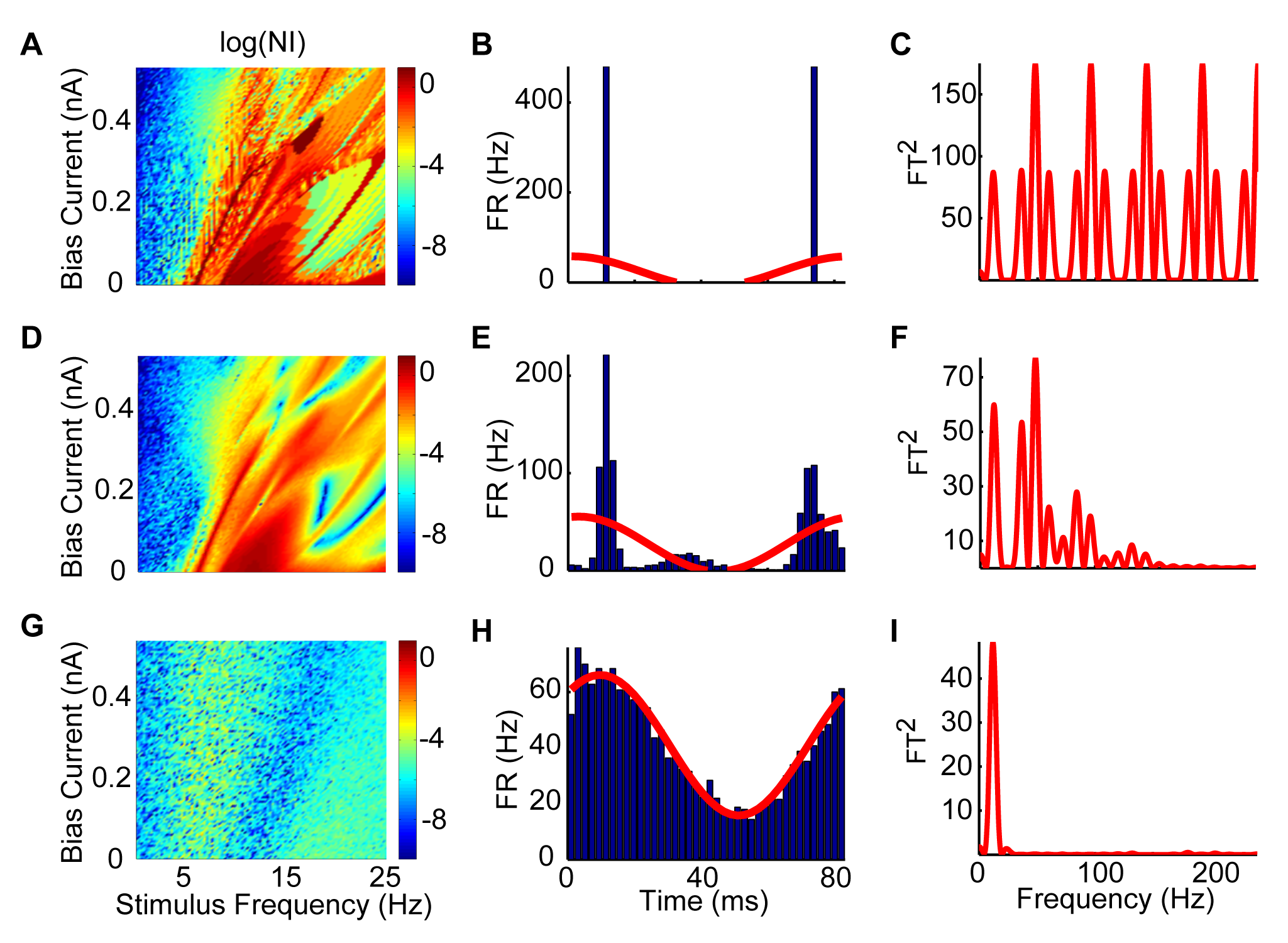

Supplement: Figure S3 — A nonlinearity index (NI) gives qualitatively similar results to those obtained with the PLI measure. A) NI as a function of the bias current and stimulus frequency without noise. B) Example PSTH responses corresponding to and . C) The squared magnitude of the Fourier transform of the PSTH response. D) NI as a function of the bias current and stimulus frequency with low intensity noise. E) Example PSTH responses corresponding to and . F) The squared magnitude of the Fourier transform of the PSTH response. G) NI as a function of the bias current and stimulus frequency with high intensity noise. H) Example PSTH responses corresponding to and . I) The squared magnitude of the Fourier transform of the PSTH response. (TIF) [file pcbi.1002120.s003.tif]

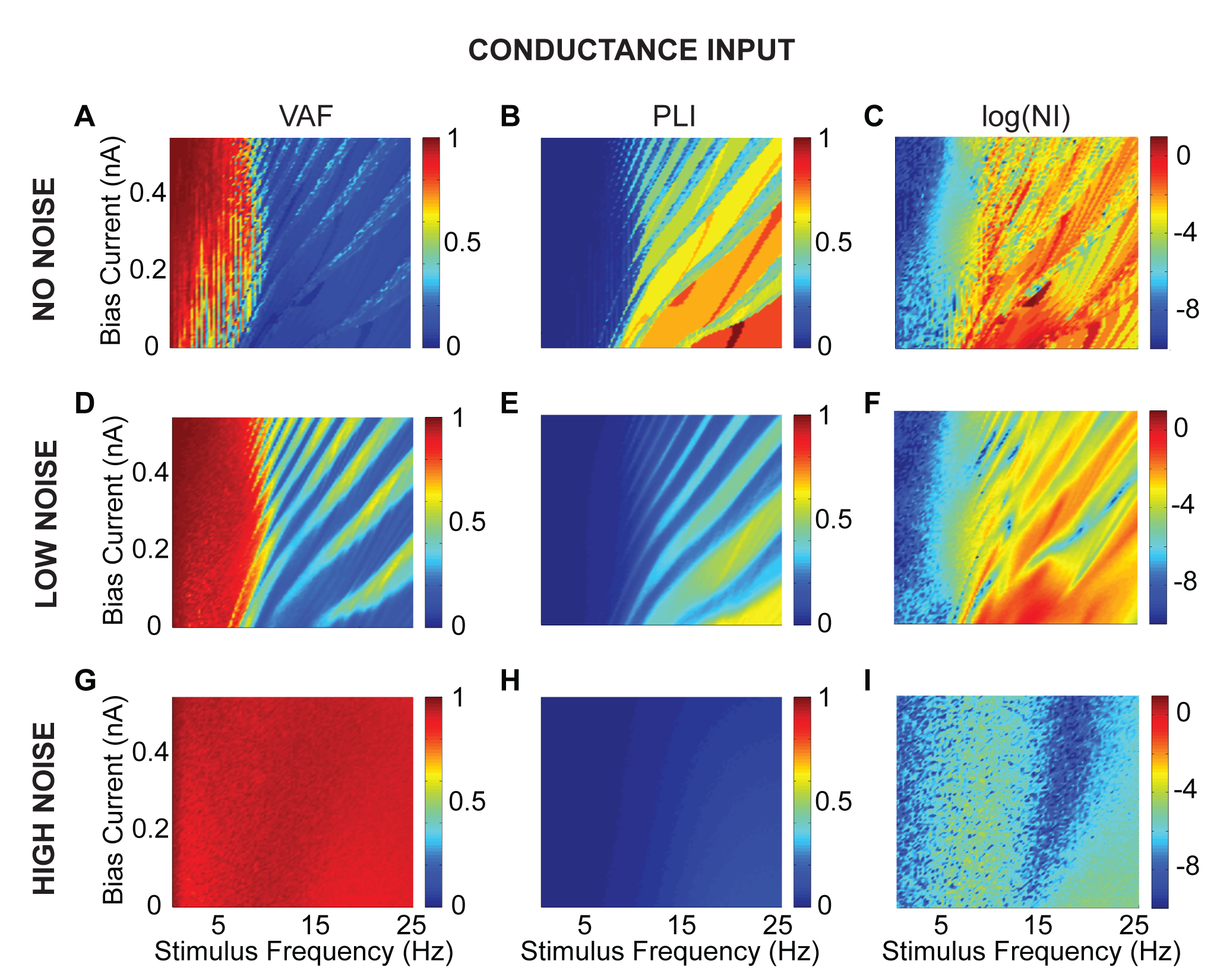

Supplement: Figure S4 — Synchronization to sinusoidal conductance input and the effects of noise. A) VAF as a function of the bias current and stimulus frequency without noise. B) PLI as a function of the bias current and stimulus frequency without noise. C) NI as a function of the bias current and stimulus frequency without noise. D) VAF as a function of the bias current and stimulus frequency with low intensity noise. E) PLI as a function of the bias current and stimulus frequency with low intensity noise. F) NI as a function of the bias current and stimulus frequency with low intensity noise. G) VAF as a function of the bias current and stimulus frequency with high intensity noise. H) PLI as a function of the bias current and stimulus frequency with high noise. I) NI as a function of the bias current and stimulus frequency with high intensity noise. All other parameters values were the same as those used in the equivalent simulations shown in Figures 6, 8, and 9 for current input, with the additional excitatory synaptic conductance . (TIF) [file pcbi.1002120.s004.tif]
